# Study of the Dielectric and Corona Resistance Properties of PI Films Modified with Fluorene Moiety/Aluminum Sec-Butoxide

**DOI:** 10.3390/polym16060767

**Published:** 2024-03-11

**Authors:** Changhai Zhang, Ziyang Liu, Chao Tang, Tiandong Zhang, Yue Zhang, Yongquan Zhang, Qingguo Chi

**Affiliations:** 1 Key Laboratory of Engineering Dielectrics and Its Application, Ministry of Education, Harbin University of Science and Technology, Harbin 150080, China; 2 School of Electrical and Electronic Engineering, Harbin University of Science and Technology, Harbin 150080, China

**Keywords:** polyimide, molecular structure control, breakdown strength, corona resistance

## Abstract

With the policy tilt and increased investment in research and development in the world, new energy vehicle technology continues to progress and the drive motor power density continues to improve, which puts forward higher requirements for the comprehensive performance of the core insulating material enameled wire enamel for drive motors. Polyimide (PI) has excellent electrical insulation properties, and heat resistance is often used to drive the motor winding insulation. To further improve the corona resistance and insulating properties of PI wire enamel varnish, in this paper, firstly, fluorene groups with a rigid conjugated structure were introduced into the molecular chain of the PI film by molecular structure modulation, and then uniformly dispersed alumina nanoclusters (AOCs) were introduced into the PI matrix by using an in situ growth process to inhibit the migration of high-energy electrons. The quantum size effect of the alumina nanoclusters was exploited to synergistically enhance the suppression and scattering of energetic moving electrons by PI-based composite films. The results show that the breakdown field strength of the PI-based composite film (MPI/1.0 vol% AOC) reaches 672.2 kV/mm, and the corona resistance life reaches 7.9 min, which are, respectively, 1.55 and 2.19 times higher than those of the initial PI film. A PI-based composite film with excellent insulating and corona resistance properties was obtained.

## 1. Introduction

Polyimide (PI) films are widely used in various types of electrical equipment insulation systems, such as drive motors for new energy vehicles, because they contain rigid structures, such as imide rings and aromatic rings, which make them resistant to high and low temperatures, ultraviolet radiation, chemical solvents, and high insulation [1,2,3,4,5,6,7,8,9]. Corona discharge is a localized self-sustained discharge of a gaseous medium in an inhomogeneous electric field, and is an early stage in the development of the breakdown process of the medium in an inhomogeneous electric field. At present, with the continuous improvement in the power density, the working conditions of new energy vehicle drive motors are more complex, such as over-voltage or under-voltage, high temperature, and other harsh conditions, resulting in the drive motor winding turn-to-turn insulation being always in a strong electric field, high-energy electrons, and high temperature in a harsh environment. It needs to withstand the heat generated by the high-speed operation of the motor, which makes the surface of enameled wire more prone to the corona discharge phenomenon and reduces the insulation strength. Under the joint action of the electric field and the heat field, its molecular chain gradually fractures and degrades, seriously degrading the insulating properties of the enameled wire film, which ultimately leads to insulation failure, restricting the efficient and reliable operation of the drive motors of new energy vehicles [10,11,12,13]. Therefore, it is of great practical significance to study and obtain PI-based wire enamels with excellent corona resistance, insulating properties, and thermal stability to promote the technological advancement of new energy vehicles and the increase in the voltage level [14,15].

The current polyimides prepared by in situ polymerization have a poor corona resistance and insulating properties at high-frequency voltages, which are unable to meet the increasing current demand for high-power density drive motors [16,17]. To improve the corona resistance and insulating properties of polyimide films, in recent years, researchers both domestically and overseas have conducted extensive studies on polyimide films with the help of inorganic nano-doping, multilayer structural design, and molecular structure modification.

The homogenized electric field and heat evacuation of PI-based composite films can be made possible by doping a large number of nanoparticles, thus improving the corona resistance of polyimide; however, due to the large specific surface area of nanoparticles, the addition of a large number of nanoparticles is very likely to cause the uneven distribution of nanoparticles, agglomeration, and other problems, resulting in film defects [18]. Feng Lang [19] prepared several PI/Al_2_O_3_ nanocomposite films that were changed using in situ polymerization and ODPA-ODA-SDA. These films demonstrated an enhanced thermal stability and, when the proportion of Al_2_O_3_ nanoparticles reached 12 wt%, a corona aging life that was 6.14 times greater than that of ODPA-ODA. Junwei Zha [20] prepared PI/TiO_2_ nanocomposite films with a good corona resistance through in situ dispersion polymerization. The paper investigated the impact of the TiO_2_ concentration and corona aging time on the films’ dielectric characteristics; the findings demonstrated that the composite films’ corona resistance increased as the TiO_2_ fillers reached 25 wt%, but the breakdown field strength of the composite films decreased from 210 MV/m to 110 MV/m. Lili Ma [21] attempted to modify and dope two-dimensional, layered double hydroxide (LDH) with potassium perfluorooctanesulfonate (PFOS) into polyimide films, and due to the incorporation of LDH with a high dielectric constant, the films were transformed into two-phase polymers containing the properties of both crystalline and amorphous regions. In addition to the enhancement in the corona resistance due to the formation of crystalline regions, the height of the potential barrier between the two phases also increases with the increase in the dielectric constant, which increases the difficulty of charge injection, and generally results in a significant improvement in the corona-resistant life of the film, up to 21 times that of pure PI. However, the doping of LDH with a high dielectric constant resulted in the degradation of the insulating properties of the PI-based composite films. Xiang Li [22] used corona-resistant fluorinated Al_2_O_3_ (F-Al_2_O_3_) to build a solid barrier for the dense SiO_2_-like storage space, which effectively inhibited the phase separation behavior of siloxane-modified PI through chain expansion co-polymerization (PI-e-Si). The resulting composite film (PI-e-Si/F-Al_2_O_3_) further improved the corona-resistant lifetime (up to 66.8 min). Xinyu Ma [23] prepared a series of PI/nano-Al_2_O_3_ composite films with a novel trilayer structure by in situ polymerization using pyromellitic dianhydride and 4,4-diamino biphenyl as raw materials, N, N-Dimethylacetamide as the solvent, and doped nano-Al_2_O_3_, and enhanced the corona resistance on the surface of the composite films by increasing the rate of iridization in conjunction with trilayer composite films. At the same time, increasing the iridization rate also improves the corona resistance time and electrical breakdown strength of the composite film.

The multilayer structure prone to interface bonding is not close; so, the dielectric loss increases and other problems, in the actual application of the process, increase the probability of equipment insulation failure. Zhiqiang Wu [24] prepared sandwich-structured films with a bottom layer made of linear dielectric polyimide (PI), a middle layer made of two-dimensional boron nitride nanosheets (BNNSs), and a top layer made of ferroelectric dielectric polyvinylidene fluoride (PVDF). High polarization was provided by the PVDF ferroelectric layer in the films, while reduced dielectric loss and enhanced dielectric breakdown strength were achieved by the PI linear layer, and a two-dimensional lamellar BNNS layer further enhanced the dielectric breakdown insulation.

Molecular structure modification leads to the enhancement in the dielectric strength of the matrix, but the nature of the different groups may cause the deterioration of the mechanical properties, thermal stability, etc., of the composite films [25,26,27,28,29,30,31]. Xuehui Peng [32] synthesized a novel polyimide copolymer (ZnTPP-PI) based on a combination of molecular engineering and co-polymerization methods by introducing zinc hyperconjugated tetraphenyl porphyrin units into the molecular backbone, which achieved an increase in the breakdown strength (216.1 kV/mm).

In summary, there are few reports on achieving the synergistic optimization of corona resistance and insulating properties of PI-based films and the compatibility of the synthesis process for industrial production. Due to the large free volume of the fluorene group and its benzene ring structure, the dielectric and insulating properties of the polymer can be optimized and the thermal stability of the polymer can be improved. In this paper, we introduced fluorene groups by changing the PI-synthesized monomer (FFDA) from improving the dielectric strength of the polyimide matrix. In order to solve the problem of the inhomogeneous distribution of the traditional inorganic particles in the matrix, we selected aluminum sec-butoxide, instead of the traditional inorganic particles for doping. The introduction of homogeneously dispersed AOCs in the PI-based composite films by using in situ growth, the effects of the modification of fluorene groups, and the doping amount of aluminum sec-butoxide on the microstructure and macroscopic properties of the PI-based composite films (MPI/AOC) were systematically investigated, and the corona resistance mechanism of the MPI/AOC composite films was systematically probed in conjunction with the results of the study. Ultimately, the MPI/1.0 vol% AOC composite film had a high breakdown field strength (672.2 kV/mm) and excellent corona resistance (7.9 min).

## 2. Synthesis and Structural Characterization

### 2.1. Materials

4,4′-Diaminodiphenyl ether (ODA, 98%), pyromellitic dianhydride (PMDA, 99%), N, N-Dimethylacetamide (DMAC, ≥99.8%), and 9,9-bis(4-amino-3-fluorophenyl)fluorene (FFDA, 98%) were purchased from Shanghai Macklin Biochemical Technology Co., Ltd., Shanghai, China. Aluminum sec-butoxide (Al(OC_4_H_9_)_3_, 97%) was purchased from Shanghai Aladdin Biochemical Technology Co., Ltd., Shanghai, China.

### 2.2. Preparation of the MPI Composite Films

Using in situ polymerization, the ratio of molar amounts of ODA added to FFDA was regulated (n(ODA): n(FFDA) = 9:1, 18:1, 36:1). Taking n(ODA):n(FFDA) = 18:1 as an example, a certain amount of ODA and FFDA was added into a three-necked flask containing DMAC. After completely dissolving ODA and FFDA in DMAC, a certain molar ratio of PMDA was added into the three-necked flask three times, and stirred in a vacuum until clarification under continued stirring for some time to obtain a transparent modified polyamide acid (MPAA) solution. Finally, the MPAA solution was uniformly coated onto glass sheets by a film coater and was subjected to continued thermal imidization in an oven at 80 °C for 2 h, 120 °C for 1 h, 220 °C for 1 h, and 350 °C for 2 h, to finally obtain the modified polyimide (MPI) composite films. A series of modified polyimide films (MPI) with different ratios, named MPI-1, MPI-2, and MPI-3, were prepared by varying the molar ratio of ODA to FFDA with the molar ratio of total diamine to dianhydride of 1:1.02.

### 2.3. Preparation of the MPI/AOC Composite Films

**Figure 1:**
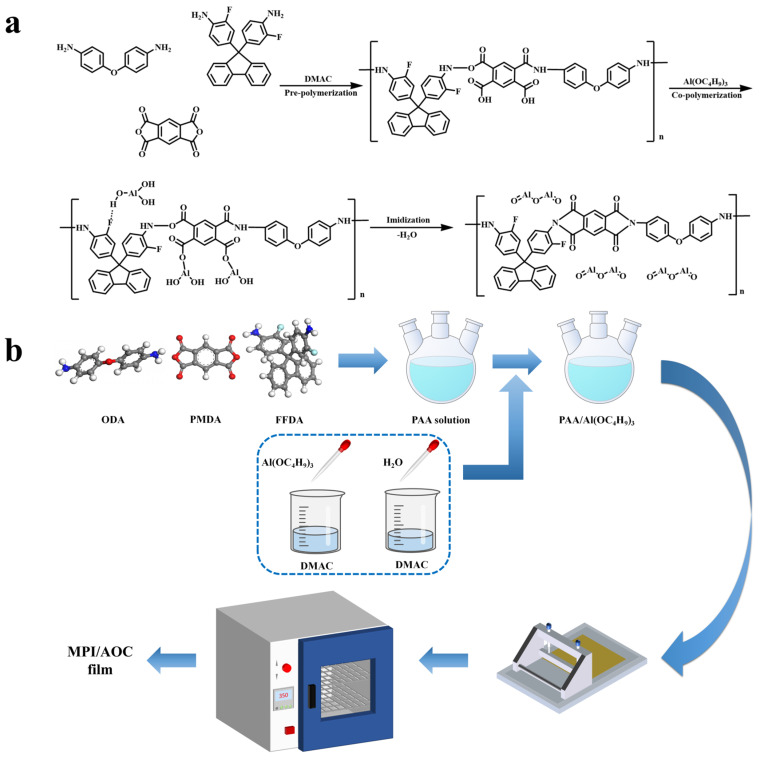
(**a**) Synthesis process and the chemical structure of MPI/AOC. (**b**) Preparation process of MPI/AOC.

The MPAA synthesis process was identical to the one described above, with the addition of the aluminum sec-butoxide (Al(OC_4_H_9_)_3_) during the in situ polymerization process, which combined the organic phase with the inorganic phase using a “positional segregation” strategy. The molecular structure and preparation process of the MPI/AOC composite films are shown in Figure 1. The added amounts of aluminum sec-butoxide (0.75 vol%, 1.0 vol%, 1.25 vol%, and 1.5 vol%) were adjusted to obtain films with optimum properties. For the example of the addition of 1 vol% AOC, 14 μL of water was dissolved in 2 mL of DMAC and 28.5 μL of Al(OC_4_H_9_)_3_ was dissolved in 3 mL of DMAC. Immediately after the synthesis of MPAA, the two prepared solutions were added dropwise, resulting in the final formation of a clear, pale yellow MPAA-AOC solution. Finally, the MPAA-AOC solution was uniformly coated on glass sheets by a film coater and was subject to continued thermal imidization in an oven at 80 °C for 2 h, 120 °C for 1 h, 220 °C for 1 h, and 350 °C for 2 h, to finally obtain the modified polyimide composite films. They were named MPI/0.75 vol% AOC, MPI/1.0 vol% AOC, MPI/1.25 vol% AOC, and MPI/1.5 vol% AOC, respectively.

### 2.4. Characterization

A scanning electron microscope (SEM) SU8020 from Hitachi, Tokyo, Japan, was used to observe the cross-section of the composite film and the morphology of the surface of the composite film after the corona breakdown. A Fourier transform infrared (FT-IR) spectrometer (EQUINOX55 from Bruker, Karlsruhe, Germany) was used to characterize the characteristic groups of the composite films, and the experimental test range was 500–4000 cm^−1^. The X-ray physical characterization of the composite films was carried out by an EMPYREAN sharp-shooting X-ray diffractometer (XRD) produced by PANARKO, Alemlo, The Netherlands, with a test voltage of 40 kV and a test current of 40 mA; the scanning range was from 2θ = 10° to 90°, the step size was 0.2°, and the time of each step was 2 s. Elemental analyses of the composite films were carried out using an ESCALAB 250Xi X-ray photoelectron spectrometer (XPS) manufactured by Thermo Scientific, Waltham, MA, USA. Small-angle X-ray scattering (SAXS) was tested and fitted to the particle size distribution of inorganic particles in the composite films using Xeuss 3.0 from Xenocs, Grenoble, France, with a focal spot diameter of 30 μm and an individual pixel size of 75 μm. The optical band gap analysis of the composite films was performed using a UV-3600i Plus (UV–vis) from Shimadzu, Kyoto, Japan. The thermal properties of the composite films were tested using a simultaneous thermal analyzer STA449 (TG) from NETZSCH, Selb, Germany, with a temperature increase rate of 20 °C/min. The mechanical properties of the composite films were tested using the Lst-10 universal material testing machine produced by Shimadzu Corporation of Kyoto, Japan. The Novocontrol Alpha-A broadband dielectric spectrum analyzer was used to test the dielectric constant and dielectric loss of the composite film, with a frequency range of 10^0^–10^6^ Hz and an electrode diameter of 9 mm. The DC breakdown characteristics of the composite films were tested using the DC breakdown module of the dielectric ferroelectric integrated test system (PolyK Technologies, Philipsburg, PA, USA), with a boosting rate of 200 V/s. The film should be placed in an oil bath during the test. This test complies with standard GB/T 1408.1-2016. The same content of the composite films was tested several times and 10 sets of valid data were taken as the experimental results. The corona resistance life was tested by an HCGF3-5 high-frequency pulse test power supply based on the GB/T22566-2017 and JB/T12421-2015 standards produced by Guilin Hecheng Electronic Technology Co. Ltd. (Guilin, China), under the conditions of room temperature, 2 kV, 15 kHz, rising edge of 100 ns, electrode diameter of 5 mm, and film test thickness of 25 μm. The components of the corona resistance test set are shown in Figure S1.

## 3. Results and Discussion

### 3.1. Characterization of the MPI Films

The chemical structure of the MPI films was characterized using infrared spectroscopy (FT-IR). As shown in Figure S2, the N-H stretching vibration at 3083 cm^−1^ and the characteristic peak near 1784 cm^−1^ correspond to the asymmetric stretching vibration of the acrylamide ring carbonyl. A symmetric stretching vibration of the two carbonyl groups on the imide ring appeared at 1726 cm^−1^, and multiple absorption peaks appeared in the range of 1100–1300 cm^−1^, which corresponded to the vibrational absorption peaks of C-F, proving that FFDA successfully accessed the PI main chain.

**Figure 2:**
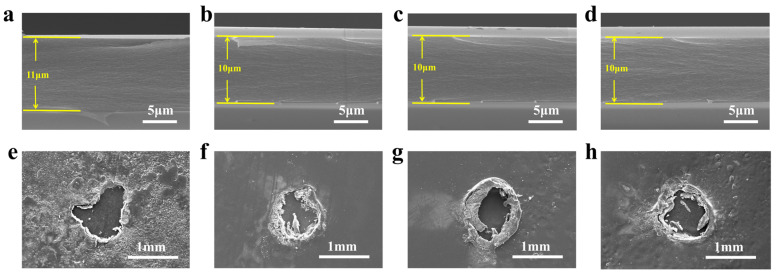
(**a**–**d**) Localized SEM images of the film sections; (**e**–**h**) surface SEM images after corona aging breakdown of PI, MPI-1, MPI-2, and MPI-3, respectively.

To analyze the effect of the microscopic morphology of fluorene group-modified MPI films before and after corona aging, scanning electron microscopy tests were first carried out on MPI film sections and film surfaces after corona aging, and the results are shown in Figure 2. From Figure 2a–d, it can be seen that the thickness of the fluorene group-modified PI films is more uniform, and the MPI films have a flat cross-sectional shape, without defects such as phase separation and holes, and have good densification, which indicates that the fluorene group-modified MPI films do not have any structural defects, and the microscopic morphology is homogeneous. Meanwhile, as shown in Figure 2e–h, after corona aging, the polymer matrix is partially eroded, and irregularly shaped corona-eroded surfaces appear, while the surface flatness of the MPI films after corona aging is higher with the increase in FFDA additions, which is attributed to the introduction of the fluorene moiety that increased the free volume of the molecular chains of the MPI films [33,34]. Thus, the energy of high-energy free electrons is weakened, and the strong electron-absorbing effect of the F atom in the fluorene group inhibits or binds the injected free electrons. The free electrons are changed from a high-energy state to a low-energy state, which reduces their attack on the molecular chain of MPI, thus improving the dielectric strength, less surface corrosion, and strong corona resistance.

### 3.2. Performance Test of the MPI Films

**Figure 3:**
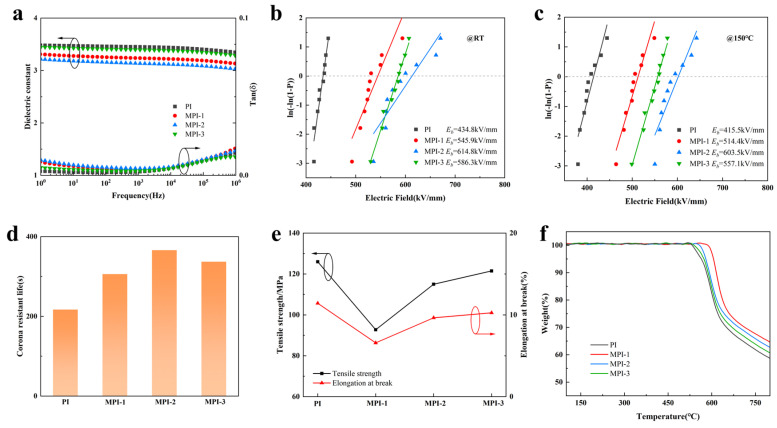
(**a**) Dielectric; (**b**) Weibull distribution of the breakdown strength at room temperature; (**c**) Weibull distribution of the breakdown strength at 150 °C; (**d**) corona resistance life; (**e**) tensile strength and elongation at break; and (**f**) TG of the PI, MPI-1, MPI-2, and MPI-3 films.

To investigate the influence law of fluorene group modification on the electrical properties of the PI-based composite films, their dielectric properties, variable temperature insulation, corona resistance, and mechanical and thermal properties were tested, and the results are shown in Figure 3. From Figure 3a, it can be seen that, with the increase in FFDA addition, the dielectric constant of the MPI films shows a trend of decreasing and then increasing, in which the MPI-2 film has the lowest dielectric constant (100 Hz, ~3.1), and there is not a vast difference in the dielectric loss at different frequencies. This is due to the introduction of a lower content of bulky fluorene groups, which disrupts the arrangement of the molecular chains of polyimide, increasing the free volume of the different molecular chains, increasing the spatial site-barrier effect, reducing the interactions between the polymer molecular chains, and decreasing the polarization rate per unit volume [35]. Secondly, the high electronegativity of the fluorine atoms reduces the induced polarization of the molecular chain, and the C-F bond itself has a low dipole polarization ability. These factors reduce the polarization ability of the MPI molecular chain, and thus reduce the dielectric constant of the MPI film. But, when too much FFDA is added, the molecular chains become entangled due to the bulk fluorene, which lowers the structure’s free volume and raises the dielectric constant [36]. As it can be seen in Figure 3b,c, the breakdown strength of the MPI-2 films is the highest, both at room temperature and at 150 °C (E_b_~614.8 kV/mm at room temperature and E_b_~603.5 kV/mm at 150 °C). This is because the strong electronegative F atoms within the fluorene group can effectively inhibit the charge migration, while the F atoms can improve the surface charge distribution of the MPI film and make the surface electric field distribution more uniform, thus enhancing the dielectric strength of the MPI film.

As it can be seen in Figure 3d, as the amount of FFDA doping in the MPI films increases, there is a trend for the corona resistance time to first grow and then decrease, and MPI-2 has the longest corona resistance lifetime (6.1 min), which improves by 1.7 times compared to the PI films. This is because the strong electronegative F atoms within the fluorene group inhibit and bind the injection of high-energy electrons, thus weakening the impact of high-energy electrons on the film and enhancing the corona resistance of the MPI film. However, when too much FFDA is added, the introduction of rigid conjugated structures, such as fluorene groups, reduces the mechanical properties of the MPI films, but they still maintain a high tensile strength (as shown in Figure 3e). Meanwhile, the conjugated structure leads to the microstructural defects of the MPI films, and the entanglement between the molecular chains enhances the polarization ability of the MPI films, which makes them more easily punctured and, to a certain extent, adversely affects their corona resistance properties [37]. To investigate the change rule of the heat resistance of the MPI composite films, a thermogravimetric analysis test was carried out, and the results are shown in Figure 3f, from which it can be seen that, with the increase in the FFDA additive content, the heat resistance of MPI is improved, for which MPI-1 has the optimal temperature resistance (~578 °C). From the point of view of the molecular structure, after the introduction of the fluorene group, the conjugated structure between the fluorene group, benzene ring, and amide ring in the MPI film prevents the rotation of the molecular chain and the conformational change, which is conducive to the improvement in the thermal stability of the MPI film.

### 3.3. Characterization of the MPI/AOC Films

**Figure 4:**
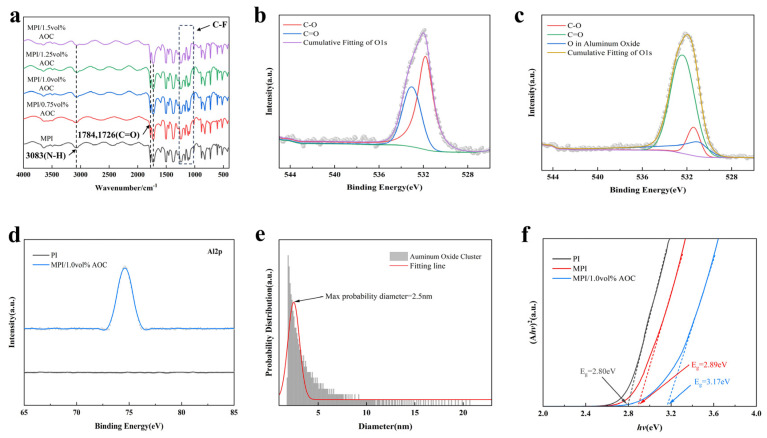
(**a**) FT-IR of MPI, MPI/0.75 vol% AOC, MPI/1.0 vol% AOC, MPI/1.25 vol% AOC, and MPI/1.5 vol% AOC; (**b**) O1s XPS spectra of PI; (**c**) O1s XPS spectra of MPI/1.0 vol% AOC; (**d**) Al2p XPS spectra of MPI/1.0 vol% AOC; (**e**) SAXS particle size distribution fitting of MPI/1.0 vol% AOC; and (**f**) UV–vis of PI, MPI-2, and MPI/1.0 vol% AOC.

The pre-polymerization of PI monomers (i.e., 4,4′-diamino diphenyl ether (ODA) and pyromellitic dianhydride (PMDA)) yields polyamic acid (PAA) containing abundant carboxyl groups, which can be used as a reactive site with the metal–alcohol precursor (i.e., aluminum sec-butoxide). In the pre-polymerization reaction, the 9,9-bis(4-amino-3-fluorophenyl)fluorene (FFDA) monomer was also utilized in this paper to introduce fluorine atoms in PAA. In this case, the freshly generated alumina nanoclusters (AOCs) can be immobilized by abundant carboxylate sites, and hydrogen bonds can be created between them and fluorine atoms, thus avoiding cluster contact and growth. The subsequent thermal imidization process separates the AOCs from the carboxyl groups, while the hydrogen bonds are broken and the AOCs are finally homogeneously dispersed in the MPI matrix. This method isolates aluminum sec-butoxide in the MPAA solution, thus solving the problem of conventional inorganic nanoparticle agglomeration. In addition to that, this method utilizes the quantum size effect [38], i.e., the electronic energy level near the Fermi energy level shifts from quasi-continuous to discrete (energy level cleavage or widening of the energy gap) when the particle size drops to a certain value, which alters the inorganic clusters’ band gap and reveals characteristics that differ from those of conventional nanoparticles. The chemical structure of the MPI/AOC composite films was characterized using infrared spectroscopy (FT-IR), and the results are shown in Figure 4a, which shows the N-H stretching vibration at 3083 cm^−1^. The characteristic peak near 1784 cm^−1^ corresponds to the asymmetric stretching vibration of the carbonyl group of the imide ring, and the symmetric stretching vibration of the two carbonyl groups on the imide ring appears at 1726 cm^−1^. Multiple absorption peaks appear in the range of 1100–1300 cm^−1^, which correspond to the vibrational absorption peaks of C-F, proving that FFDA successfully accessed the PI backbone. In Figure S3, the polyimide-insulating dielectrics all show broad peaks from 10° to 30°, which indicates that the polyimide composite films are amorphous. As shown in Figure S7, the XPS full spectrum test of the PI film and MPI/1.0 vol% AOC film revealed that the peak positions of F1s and Al2p appeared in the spectrum peaks of MPI/1.0 vol% AOC, which also indicated the successful access of FFDA with the addition of aluminum sec-butoxide. To investigate the substances generated by the addition of aluminum sec-butoxide, the O1s and Al2p in Figure S7 were subjected to fine spectral peak splitting. Figure 4b,c shows the XPS fine spectra of PI, MPI/1.0 vol% AOC O1s, which reveals that the peaks of O in alumina appear in the composite film of MPI/1.0 vol% AOC. Figure 4d shows the XPS fine spectrum of PI, MPI/1.0 vol% AOC Al2p, which reveals that the MPI/1.0 vol% AOC film has an Al peak position at 74.6 eV; this, combined with the O1s and Al2p results of the XPS test, suggests that the aluminum sec-butoxide introduced during the synthesis is transformed into alumina nanoclusters through the thermally imidized process and is uniformly dispersed into the MPI films. As shown in Figure 4e, the internal inorganic particle size of the MPI/1.0 vol% AOC composite films was tested and fitted statistically using SAXS, assuming that the alumina inorganic clusters have a spherical structure, and the calculations showed that the maximum possible distribution of the particle sizes was 2.5 nm in the MPI/1.0 vol% AOC composite films. To analyze the effect of fluorene group modification with the introduction of AOCs on the forbidden bandwidth of the PI-based films, UV–vis tests were carried out on three kinds of films, namely PI, MPI-2, and MPI/1.0 vol% AOC, and the results are shown in Figure 4f. As it can be seen from the figure, the introduction of FFDA results in a larger forbidden bandwidth (~2.89 eV) of the MPI film due to the electrophilic ability of the F atoms in the fluorene moiety, which can lead to the excellent insulating properties of the MPI film. On this basis, the bandwidth becomes further larger (~3.17 eV) in the films to which 1 vol% of aluminum sec-butoxide was introduced, indicating that both the fluorene group modification and the uniformly dispersed alumina nanoclusters enhance the insulating properties of the PI-based films. In addition, the cutoff edge of the MPI/1.0 vol% AOC film begins to bend at longer wavelengths compared to that of the MPI film, suggesting that there are new energy levels in the bandgap of the MPI/AOC film to absorb photons with lower energies, which enhances the film’s ability to trap and bind high-energy free electrons.

**Figure 5:**
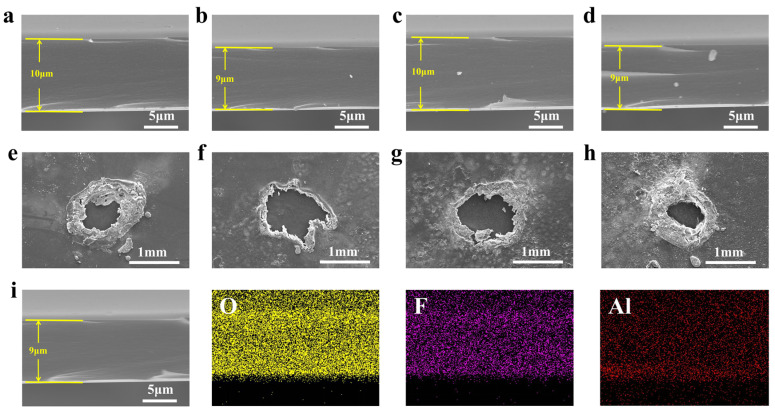
(**a**–**d**) Localized SEM images of film sections, (**e**–**h**) surface SEM images after corona aging breakdown of the MPI/0.75 vol% AOC, MPI/1.0 vol% AOC, MPI/1.25 vol% AOC, and MPI/1.5 vol% AOC films, and (**i**) mapping images of film sections of the MPI/1.0 vol% AOC film.

**Figure 6:**
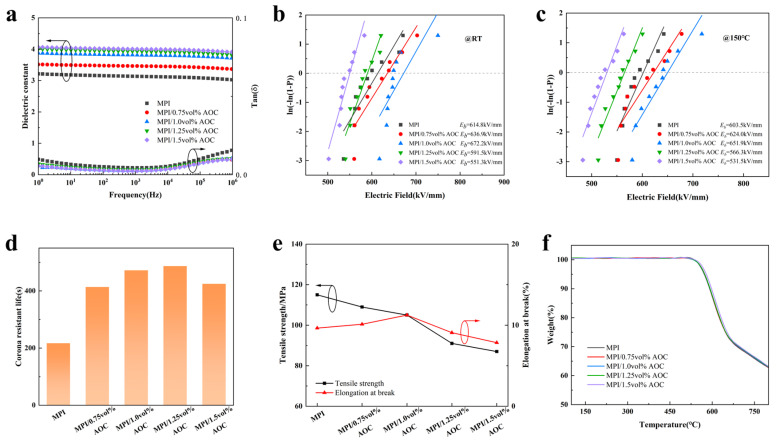
(**a**) Dielectric; (**b**) Weibull distribution of the breakdown strength at room temperature; (**c**) Weibull distribution of the breakdown strength at 150 °C; (**d**) corona resistance life; (**e**) tensile strength and elongation at break; and (**f**) TG of the MPI, MPI/0.75 vol% AOC, MPI/1.0 vol% AOC, MPI/1.25 vol% AOC, and MPI/1.5 vol% AOC films.

The cross-section structure of the MPI/AOC films and their surface structure after corona aging were tested by SEM, and the results are shown in Figure 5. As it can be seen in Figure 5a–d, almost no bulky alumina nanoclusters appear in the MPI/AOC films doped with 0.75 vol% and 1.0 vol% of aluminum sec-butoxide, which is different from conventional inorganic particle doping in that it solves the dispersibility problem associated with conventional inorganic particle doping, allowing the inorganic particles to be uniformly dispersed in the MPI. Due to the immobilization of AOCs by carboxyl and hydrogen bonding, even though a larger volume of nanoclusters (20 nm, Figure 6b) was generated, they can be distributed more uniformly in the MPI/AOC film. A small number of visible alumina clusters appeared in the MPI/AOC films when the doping content of aluminum sec-butoxide was 1.25 vol%, and more visible alumina nanoclusters appeared in the MPI/AOC films when the doping content of aluminum sec-butoxide reached 1.5 vol%. On this basis, tests were conducted to characterize the surface morphology of the MPI/AOC films after corona aging, and the results are shown in Figure 5e–h. It can be seen from the figure that the number of inorganic particles precipitated on the surface of MPI/AOC films after corona breakdown gradually increases with the increase in the AOC content. To further investigate the distribution of AOCs in the MPI/AOC films, mapping tests were carried out on the elements of the film sections, and the results are shown in Figure 5i. From the mapping diagrams of the MPI/AOC film sections, it is seen that the element Al is uniformly distributed in the film sections, which proves that the use of carboxyl groups with hydrogen bonding to immobilize aluminum sec-butoxide and uniformly disperse the AOCs into the MPI/AOC films is feasible and effective.

### 3.4. Performance Test of the MPI/AOC Films

As shown in Figure 6a, the dielectric constant gradually increased with the increase in the aluminum sec-butoxide content, which was attributed to the fact that the content and particle size of the alumina clusters in the MPI/AOC films increased with the increase in the aluminum sec-butoxide content. The interfacial polarization effect between the high-dielectric-constant alumina clusters and the PI matrix led to the elevated dielectric constant of the MPI/AOC films, but the dielectric constant and dielectric loss of the composite films remained at a low level.

Since free electrons are ultimately responsible for both electrical and thermal breakdowns, the trapping of free electrons by the MPI/AOC films is also reflected in the breakdown strength [39,40]. In Figure 6b,c, the breakdown field strengths of the composite films were tested at room temperature and 150 °C, and the results show that the breakdown strengths firstly increase and then decrease with the increase in the AOC content, and the MPI/1.0 vol% AOC films have the highest breakdown field strengths at both room temperature and 150 °C (E_b_~672.2 kV/mm at room temperature and E_b_~651.9 kV/mm at 150 °C). This is due to the more uniform distribution of alumina nanoclusters compared to the discrete distribution of alumina nanoparticles, which can more effectively block the impact of the injected high-energy free electrons at the electrodes and enhance the breakdown strength of the MPI/AOC films. The corona resistance time of MPI/AOC films doped with different contents of aluminum sec-butoxide is shown in Figure 6d, from which it can be seen that the corona resistance time of the MPI/AOC films first rises and then decreases with the increase in the AOC content. MPI/1.25 vol% AOC has the longest corona resistance life (8.1 min), with MPI/1.0 vol% AOC having a corona resistance life of 7.9 min, which is a 2.19 times improvement compared to PI films. This is because, as the doping of alumina clusters increases, the number of energetic particle sites inside the MPI/AOC film that block corona generation increases, and this increase is positively correlated with the corona resistance lifetime of the film. At the same time, the introduction of nanoclusters also facilitates charge migration, which can improve the local charge aggregation due to defects and other factors, thus homogenizing the electric field and reducing the risk of the corona breakdown of the MPI/AOC films. However, when the doping amount of aluminum sec-butoxide is further increased to 1.5 vol%, the film has more bulky alumina clusters inside the film, which tends to cause structural defects, leading to a decrease in the corona breakdown resistance time of the film. Meanwhile, as shown in Figure 6e, with the increase in aluminum sec-butoxide doping, the tensile strength of the MPI/AOC films shows a decreasing trend, and the elongation at break first rises and then decreases, but still maintains more excellent mechanical properties. Finally, the thermal weight loss of the PI-based films was tested, and the results in Figure 6f show that the temperature resistance of the films is basically unchanged with the increase in the doping amount of aluminum sec-butoxide, and the thermal decomposition temperature is maintained at about 528 °C, i.e., the elevation of the content introduced to the alumina clusters does not decrease the thermal stability of the films, which indicates that the aluminum sec-butoxide is transformed into alumina, stabilized at this time in the MPI/AOC composite films.

### 3.5. Analysis of the Corona Resistance Mechanism

**Figure 7:**
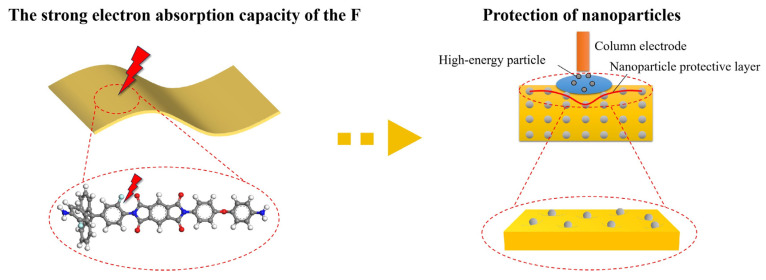
MPI/AOC corona resistance mechanism.

In the corona resistance test, the column electrode emits high-energy particles, and a partial discharge phenomenon occurs on the surface of the PI film in contact with it. The corona resistance mechanism of the PI/AOC composite film is shown in Figure 7. High-energy electrons in the PI will shift from high-energy to low-energy states after being captured [41]. While the energy difference will be transferred to other electrons and turn them into hot electrons, this process will produce free radicals and start a chain reaction of free radicals when it reacts with the polyimide molecule, leading to polymer degradation and ultimately the formation of insulation weak zones until the breakdown of aging. Fluorene groups, F atoms, and alumina clusters have been introduced into MPI/AOC composite films, and the F atoms and alumina clusters can improve the distribution of charge on the MPI surface, resulting in a more uniform distribution of the electric field on the MPI surface. At the same time, the corona resistance of the film is improved because the free volume of the molecular chain becomes larger and the strong electron-absorbing ability of the F atom reduces the energy of the free electrons. In addition, when the substrate surface is eroded by corona, alumina clusters precipitate and form a dense protective layer on the surface of the composite film, and the inorganic particles contribute to the charge migration diffusion and have the role of heat conduction [42]. Therefore, it can play the role of homogenization of the electric field [43] and reduce the damage of the thermal field on the film, the integrated protection of the internal structure of the material, and ultimately MPI/AOC composite films are obtained with excellent insulating properties and corona resistance.

## 4. Conclusions

In this study, a polyimide composite material fabricated by the modification of organic molecules combined with the trace doping of inorganic nanoparticles was designed to synergistically endow the PI film with excellent dielectric and corona resistance properties. First, the introduction of fluorene groups and F atoms via the monomer FFDA was conducted. When ODA:FFDA = 18:1, the breakdown field strength of the MPI-2 composite film reached 614.8 kV/mm and the corona resistance time reached 6.1 min. Subsequently, aluminum sec-butoxide was added to this system, resulting in the homogeneous dispersion of the AOCs in the polymer matrix without the need for any auxiliary dispersing steps. The insulating properties and corona resistance of the PI films were optimized by synergistically enhancing the inhibition of electrons using the quantum size effect. Finally, the MPI/1.0 vol% AOC composite films had excellent dielectric properties (a lower dielectric constant and dielectric loss), with a breakdown field strength of 672.2 kV/mm and a corona resistance life of 7.9 min, which are 1.55 and 2.19 times higher than those of the pure PI. The synthesis method of this material provides a new way to synergistically improve the insulation and corona resistance of the polymer and this material has a broad application prospect in the fields of new energy vehicles, aerospace, microelectronic, and other fields, as it is an important insulating material.

## Data Availability

Data are contained within the article and Supplementary Materials.

## Supplementary Materials

The following supporting information can be downloaded at: https://www.mdpi.com/article/10.3390/polym16060767/s1, Figure S1: High frequency corona aging test stand. Figure S2: Infrared spectra of PI, MPI-1, MPI-2, MPI-3. Figure S3: XRD of MPI, MPI/0.75 vol% AOC, MPI/1.0 vol% AOC, MPI/1.25 vol% AOC, MPI/1.5 vol% AOC. Figure S4: SAXS of the MPI/1.0vol AOC. Figure S5: SAXS profiles of MPI/1.0 vol% AOC. Figure S6: UV-vis of PI, MPI, MPI/1.0 vol% AOC. Figure S7: XPS of PI, MPI/1.0 vol% AOC.
