# Supplementary material for: Study of the Dielectric and Corona Resistance Properties of PI Films Modified with Fluorene Moiety/Aluminum Sec-Butoxide
Source: Polymers (Basel). 2024 Mar 11;16(6):767. doi: 10.3390/polym16060767 (PMC10975545; doi:10.3390/polym16060767)
Supplement: Supplementary file 1 [file polymers-16-00767-s001.zip › polymers-2883120-supplementary.pdf]

Supporting Information

Study of the Dielectric and Corona Resistance Properties of PI Films Modified with Fluorene Moiety/Aluminum Sec-Butoxide

Changhai Zhang [1,2], Ziyang Liu [1,2], Chao Tang [1,2,*], Tiandong Zhang [1,2], Yue Zhang [1,2], Yongquan Zhang [1,2] and Qingguo Chi [1,2,*]

[1] Key Laboratory of Engineering Dielectrics and Its Application, Ministry of Education, Harbin University of Science and Technology, Harbin 150080, China

[2] School of Electrical and Electronic Engineering, Harbin University of Science and Technology, Harbin 150080, China

* Correspondence: chaotang@hrbust.edu.cn (C.T.); qgchi@hrbust.edu.cn (Q.C.)

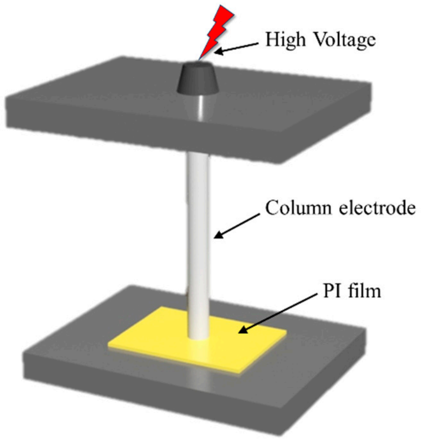

Figure S1 High frequency corona aging test stand.

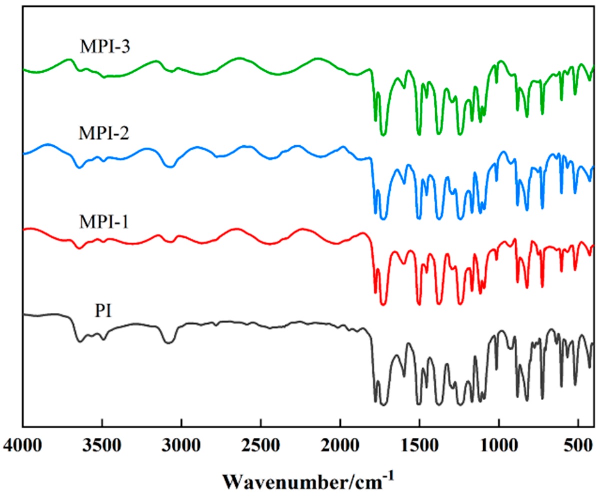

Figure S2 Infrared spectra of PI, MPI-1, MPI-2, MPI-3.

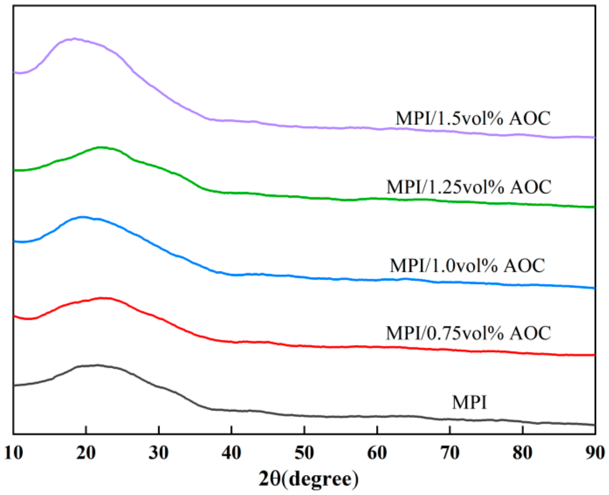

Figure S3 XRD of MPI, MPI/0.75 vol% AOC, MPI/1.0 vol% AOC, MPI/1.25 vol% AOC, MPI/1.5 vol% AOC.

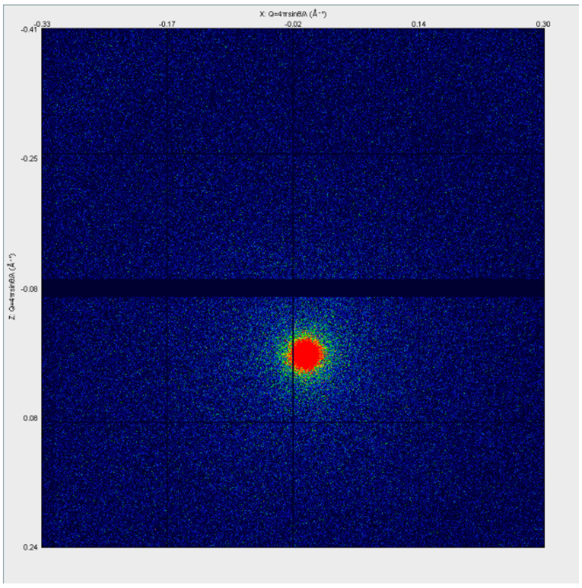

Figure S4 SAXS of the MPI/1.0vol AOC.

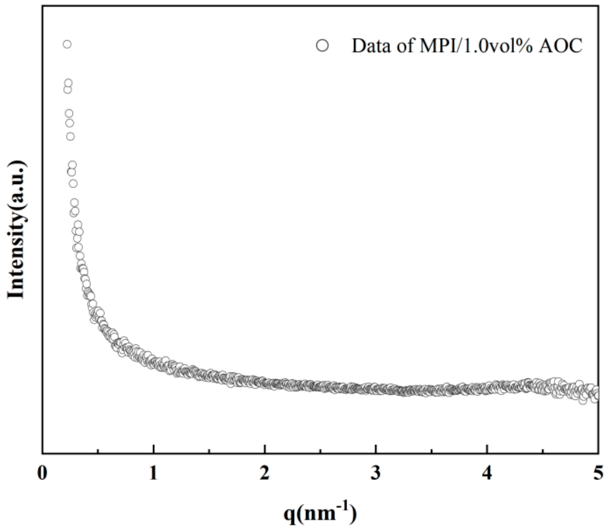

Figure S5 SAXS profiles of MPI/1.0vol% AOC.

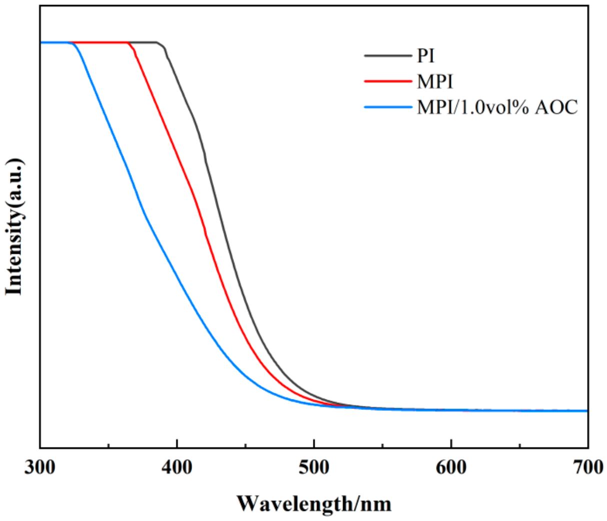

Figure S6 UV-vis of PI, MPI, MPI/1.0vol% AOC.

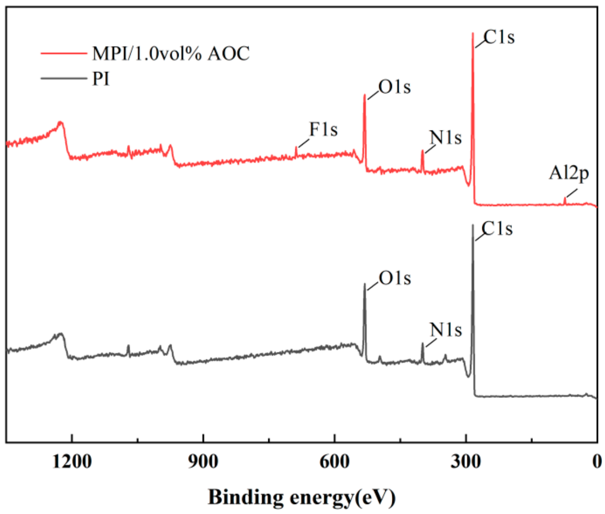

Figure S7 XPS of PI, MPI/1.0vol% AOC.
